# Using spatial equity analysis in the process evaluation of environmental interventions to tackle obesity: the healthy towns programme in England

**DOI:** 10.1186/1475-9276-12-43

**Published:** 2013-06-17

**Authors:** Alice M Dalton, Andrew Jones, David Ogilvie, Mark Petticrew, Martin White, Steven Cummins

**Affiliations:** 1 School of Environmental Sciences, University of East Anglia, Norwich Research Park, Norwich, UK; 2 UKCRC Centre for Diet and Activity Research (CEDAR), Institute of Public Health, Cambridge, UK; 3 Norwich Medical School, University of East Anglia, Norwich Research Park, Norwich, UK; 4 Medical Research Council Epidemiology Unit, Institute of Public Health, Cambridge, UK; 5 Department of Social and Environmental Health Research, London School of Hygiene and Tropical Medicine, London, UK; 6 Fuse, UKCRC Centre for Translational research in Public Health, Institute of Health & Society, Newcastle University, Newcastle upon Tyne, UK

**Keywords:** Spatial inequalities, Healthy towns, Process evaluation, Intervention

## Abstract

**Introduction:**

Process evaluations of environmental public health interventions tend not to consider issues of spatial equity in programme delivery. However, an intervention is unlikely to be effective if it is not accessible to those in need. Methods are required to enable these considerations to be integrated into evaluations. Using the Healthy Towns programme in England, we demonstrate the potential of spatial equity analysis in the evaluation of environmental interventions for diet and physical activity, examining whether the programme was delivered to those in greatest need.

**Methods:**

Locations of new physical infrastructure, such as cycle lanes, gyms and allotments, were mapped using a geographic information system. A targeting ratio was computed to indicate how well-located the infrastructure was in relation to those at whom it was specifically aimed, as detailed in the relevant project documentation, as well as to generally disadvantaged populations defined in terms of UK Census data on deprivation, age and ethnicity. Differences in targeting were examined using Kruskal-Wallis and t-tests.

**Results:**

The 183 separate intervention components identified were generally well located, with estimated targeting ratios above unity for all population groups of need, except for black and ethnic minorities and children aged 5–19 years. There was no evidence that clustering of population groups influenced targeting, or that trade-offs existed when components were specifically targeted at more than one group.

**Conclusions:**

The analysis of spatial equity is a valuable initial stage in assessing the provision of environmental interventions. The Healthy Towns programme can be described as well targeted in that interventions were for the most part located near populations of need.

## Background

Effective public health interventions should narrow inequalities by having a positive impact on the health of populations most in need. However, this often does not occur, and interventions may sometimes widen inequalities [1]. These intervention generated inequalities may be more pronounced for certain classes of intervention, such as those reliant on voluntary behaviour change, which may result in lower uptake among more disadvantaged groups [2]. This has been shown in interventions such as those to promote breast feeding [3], seatbelt use [4] and cancer screening [5]. Furthermore, the effectiveness of interventions may be socially patterned, as found by studies of smoking restrictions [6] and bicycle helmet legislation [7]. These observations are of particular concern given the potential existence of ‘deprivation amplification’ [8], whereby the most socioeconomically disadvantaged experience poorer health and poorer access to resources [9]. A version of the ‘inverse care law’ [10] may therefore operate for many interventions, whereby the provision of facilities that are spatial in nature may be poorest in areas with greatest need. One way of investigating such issues within evaluations is to explore the underlying geographical distribution of resources in relation to the context of the areas in which they are provided [11].

Achieving social equity in environmental planning and decision making processes is increasingly recognised as an important component of environmental policies in many countries [12]. Building on the concept of environmental inequity in the USA – defined as the apparent unequal geographical distribution of benefits or burdens among those in poverty or for minority groups [13] – early work focused on consideration of disamenities such as hazardous facilities [14,15]. Subsequently, access to amenities has been considered, with studies examining the locations of parks [16], playgrounds [17,18] and public health facilities [19], often suggesting that disadvantaged populations may have poorer access to given resources. A valuable component of environmental equity is therefore that of spatial equity.

An important current area of research is the exploration of the provision of resources that support physical activity. People who engage regularly in physical activity are less likely to experience a range of preventable chronic conditions including obesity [20]. Consequently, interventions have been developed in order to increase activity levels in target groups or populations [21] with mechanisms including the use of information, targeted behavioural and social programmes, and changing the physical environment and planning policy associated with it [22]. For example, interventions to encourage walking and cycling have included publicity campaigns to increase awareness, financial incentives, and improvements to footpaths and cycle routes [21]. Disadvantaged groups, such as those with low socio-economic status and ethnic minorities, often have higher levels of obesity and have sometimes been shown to live in areas with poorer access to facilities for physical activity or healthier eating [23]. This emphasises the importance of understanding how need and provision vary spatially so that interventions can be located to serve these high-need yet often overlooked populations. Nevertheless, decisions about where to locate infrastructure can be difficult to reconcile with the location of target populations due to issues of land availability and other context-specific circumstances [24]. There remains a paucity of evidence regarding the delivery of effective interventions [25] due to limited evaluation of interventions [26].

Methods using geographic information system (GIS) technology may be employed to assess the spatial equity of amenities, resources and infrastructure. Studies have calculated the distances between residential locations and amenities [27] and the number of facilities available per capita [28]. However, the utility of these findings is limited, as not all have considered the underlying geography of need, so it is not always known if a facility favours or disadvantages a certain type of population [17]. Some studies have addressed this; Nicholls [17,29] evaluated the locations of disadvantaged populations (non-white populations, children, economically disadvantaged) with and without access to parks within 800m of home, finding that parks tended to be located in areas with greatest need. Such literature is generally concerned with the location of existing infrastructure rather than the evaluation of recent modifications to the built environment. Nevertheless, method and learning from these studies may be used to inform the evaluation of infrastructural interventions to target population health behaviours.

Process evaluation is central to determining whether interventions perform as intended [30] and can therefore aid understanding of how the context within which they are developed may affect their eventual effectiveness [31]. Spatial equity is the first step in a process towards reducing health inequality via structural or area-based interventions and should therefore be evaluated accordingly. If the intervention does not achieve a basic level of ‘availability’ through accurate targeting, then the next steps - uptake, efficacy, long-term compliance and health outcomes [1] - are unlikely to be achieved. It is thus important to examine the potential reach of interventions [32], and the implications of their presence [33] in order to give insight and possible explanation for the outcomes [34]. UK guidance recommends that components of planning, implementation and operation should be reviewed as a necessary precursor to a full evaluation of health interventions [35] to understand the local context, at whom a given intervention is aimed, and the components of an intervention [34]. It has been suggested that evaluations might be further strengthened by integration of geographic data [36] and we argue this is particularly so for spatial equity analysis. This may ultimately be used to give an early indication that an intervention might not eventually be effective because it is poorly targeted.

In this paper we develop and apply methods to evaluate how the spatial location of infrastructure relates to the underlying geography of population need, using the case study of a recent government-funded programme in England, ‘Healthy Towns’ [37]. This government-funded programme aimed to provide interventions to encourage dietary and physical activity behaviour change to combat obesity. We investigate whether infrastructure developed from this was best located in relation to areas of need, according to the socio-demographic characteristics of neighbourhoods.

## Methods

### The healthy Towns programme in England

The Healthy Towns programme was funded from the £30 million ‘Healthy Community Challenge Fund’ (HCCF), set up by the UK government as part of the ‘Healthy Weight, Healthy Lives’ strategy for England [38]. This fund was allocated between nine local areas in England (Table 1), each of which had submitted a successful competitive bid for the provision of innovative interventions and community-driven programmes aimed at increasing physical activity and improving diet using a ‘whole-town’ approach [39]. The HCCF envisaged that part of the funding would enable each local area to provide a physical environment appropriate to encouraging healthy behaviours, using modifications to influence walkability, safety, vibrancy or supportiveness of the environment for active travel [40]. The areas responded by planning and implementing (building or improving) or mapping various pieces of physical infrastructure to modify the built environment and provide facilities to encourage behaviour change [39]. These interventions varied widely in nature from cycle signage and foot paths to allotment plots and green spaces.

**Table 1:** Characteristics of the local areas of the healthy towns programme

**Local areas**	**Type**^**a**^	**Population range**^**a**^	**Size (km**^**2**^**)**^**b**^	**DH funding (£M, approx)**^**a**^
Sheffield, Manchester^c^, Tower Hamlets, Dudley, Portsmouth	Large town or city	195,000 – 500,000	22 - 121	3.1 - 4.9
Middlesbrough, Halifax	Mid-size town	82,000 – 132,000	29 - 36	2.0 - 4.1
Tewkesbury, Thetford	Small town	17,000 – 22,000	4 - 6	0.9 - 1.2

Source: ^a^Department of Health [39]^b^Office for National Statistics [41]; ^c^Manchester is not included in the work presented in this manuscript, as no physical infrastructure was developed there. *Km* kilometre. *£M* pounds sterling in millions.

To assess the spatial equity of the delivery of these interventions, it was necessary to identify the spatial extent of the infrastructural developments in relation to the distribution of key population groups. This was examined to identify if particular groups were advantaged or disadvantaged in terms of local provision. In addition, funding allocations per town and by population diversity were examined to see if successful delivery of interventions varied according to the funding context. The evaluation considered the possible existence of trade-offs, whereby interventions aimed at more than one population group may have favoured one group at the expense of others in a given location.

This study is part of the wider national evaluation of the Healthy Towns programme carried out by a team including the authors of this paper. The team brings together academics with expertise in geography, public health and the evaluation of health interventions.

### Spatial location of infrastructure

All spatial analyses were conducted using GIS software (ArcGIS 9.3™ [42]). Each local area was defined by urban settlement area boundaries identified by the UK Ordnance Survey (OS), the national mapping agency of the UK. These boundaries were supplemented with a 400 m buffer in order to include populations within a short distance of the urban fringe.

Locations of physical infrastructure were identified and obtained from the Healthy Towns database developed as part of the overall programme evaluation. The database consisted of information extracted from reports and documentation provided by programme managers from each local area. This was augmented with further information from local area-specific websites, council maps, and planning applications. Updated maps and information were then reviewed by the relevant programme managers, and were amended where appropriate. Only infrastructure that was confirmed as complete or in progress was included.

Each item of infrastructure was classified according to four categories based on the primary function of that intervention: active travel (infrastructure to promote walking or cycling to access a location), food systems (outlets and facilities for growing and eating healthy food), healthy lifestyles (behaviours which positively influence health), and physical activity (movement and exercise). Classification by the type of facility provided was also undertaken. Interventions included advice/information, cafe/food co-ops, facilities for cycling/walking (e.g. cycle parking), food growing, green gym/dance studios, outdoor play area/green spaces, walking/cycling routes (e.g. paths and crossings), and walking/cycling mapping/signage. Each facility was mapped as a point, line or area as appropriate. Some components had multiple facilities that could be classified into different types or different categories. In such cases, each facility was considered separately.

**Figure 1:**
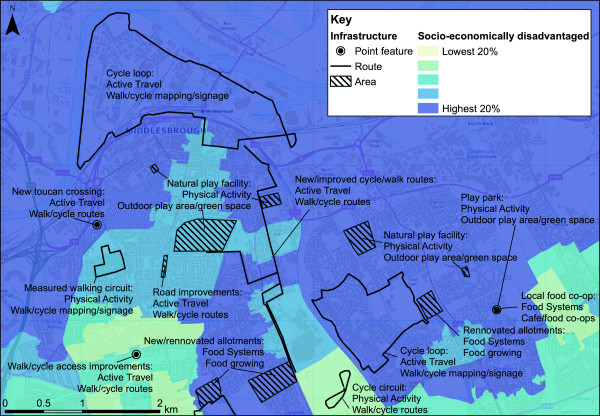
**Example of healthy towns-funded physical infrastructure, Middlesbrough.** Example of Healthy Towns-funded physical infrastructure, Middlesbrough, displayed on quintiles of socio-economic disadvantage (socio-economic classification according to NS-SEC [43]).

Figure 1 illustrates the classification of infrastructure with the example of an area of Middlesbrough. This area is categorised according to socio-economic disadvantage relative to the rest of England; the percentage unemployed and in lower quality employment according to the National Statistics Socio-economic Classification [43].

### Defining populations

Two populations of need were identified for each intervention. Firstly, specific target populations were identified according to the group(s) specified for each intervention in each area’s project initiation document. These were varied and included, for example, black and minority ethnic (BME) groups, children (various ages specified), inactive/overweight individuals and retired households. Each intervention was aimed at between one and six target populations. In order to identify the geographical distributions of these populations, data were obtained from the 2001 Census of Population in England and mapped to the smallest possible spatial unit: the Output Area (each containing an average of 297 individuals). Estimates of obesity were only available at the coarser Middle Super Output Area level (each containing an average of 7,247 individuals) [44]. The data used to define the populations are detailed in Table 2.

**Table 2:** Data used to define population

**Population of need according to healthy towns database**	**Definition used for analysis**^**a**^
All people	Total resident population
Black and minority ethnic (BME)	Total number of non-white British people (white Irish, white other, mixed, Asian or Asian British, black or black British, Chinese or other)
Children	Total resident population aged 0 – 18 years
Children/youths [specific ages]	Total resident population by appropriate age categories
Disabilities and/or learning difficulties	Total resident population with limiting long-term illness, health problems or disability
Employers and employees	Total workplace population
Families	Total number of families in households with one or more dependent children (0 – 15, or 16–18 and in full time education)
Households living in social housing	Total number of households living in social rented accommodation
Inactive/overweight	Model based estimates for percentage obese, converted to number of people based on the proportion of adults aged 16 and over in the resident population
Over 50’s	Total resident population aged 50 and above
Resident adults	Total resident population aged 18 and above
Retired households	Total number of households with pensioners (one person; one family and no others, all pensioners; other households, all pensioner)
Single parent families	Total lone parent households with dependent children (0 – 15, or 16–18 and in full time education)
Socio-economic disadvantage	Total number of people in National Statistics Socio-Economic Classifications (NS-SEC) semi-routine occupations, routine occupations, never worked and long-term unemployed

^a^Source for all datasets is Office for National Statistics [45] except for the measure for Inactive/overweight which is Office for National Statistics [46].

To enable a broader examination of equity in intervention provision we also evaluated each intervention in terms of the distribution of generally disadvantaged population groups. After reviewing evidence from the equity literature [23,47-49] and considering the target populations identified by each Healthy Town, these were determined to be four population groups comprising BME groups, retired households, all children (aged 0 – 18), and the socio-economically disadvantaged. These groups were also identified spatially and defined as the total count of people or households present in each of the four groups, using the relevant data listed in Table 2.

### Equity analysis

Analysis was conducted to assess the equity of infrastructural provision from the Healthy Towns programme in relation to the locations of each population of need. Firstly, populations with ‘good’ access to infrastructure were spatially identified. Good access was defined as living within a ten minute walk (represented by a straight line distance of 800 m) of facilities, a distance used in previous analyses of accessibility based on how far people are willing to walk to access services [29,50-52]. For route infrastructure, a distance of 100 m either side of the centreline was used to capture characteristics of populations through which the route passed.

The number of people with good access in each of the populations of need (defined as the target populations and generally disadvantaged populations) was estimated by comparing census data boundaries containing population counts with the boundaries delineated around the infrastructure (the 800 m or 100 m distance as defined above). For each group, the number of individuals or households falling within the boundaries for all interventions was then estimated, and these groups were defined as having good access. In cases where a census area was only partially classified as having good access, populations were estimated based on the size of the area of overlap. This procedure was undertaken for each population of need group, as well as the remaining population group of the town. It allowed ‘targeting ratios’ to be computed using the formula in Equation 1 to assess whether interventions were well-located for the different groups. A targeting ratio above unity indicates that the population of need were more likely to live within areas classed as ‘good access’ compared to the rest of the population within the town, whereas a value below 1 means that the population of need were spatially disadvantaged. Associated 95% confidence intervals were calculated.

(1) $$\mathrm{Targetting}\;\mathrm{ratio}=\frac{\left(\frac{\mathrm{Population}\;\mathrm{of}\;\mathrm{need}\;\mathrm{with}\;\mathrm{good}\;\mathrm{access}}{\mathrm{Population}\;\mathrm{of}\;\mathrm{need}\;\mathrm{in}\;\mathrm{the}\;\mathrm{town}}\right)}{\left(\frac{\mathrm{Rest}\;\mathrm{of}\;\mathrm{population}\;\mathrm{with}\;\mathrm{good}\;\mathrm{access}}{\mathrm{Rest}\;\mathrm{of}\;\mathrm{population}\;\mathrm{in}\;\mathrm{the}\;\mathrm{town}}\right)}$$

Funding for each Healthy Town was examined to investigate resources allocated in relation to the number of people classified as having good access to interventions. Towns were grouped into three categories of population size based on natural breaks (small 17-22 K, medium 82 -132 K, large 195-500 K), and the average amount of funding for the towns in each category was calculated according to the total disadvantaged population and the disadvantaged population with good access.

The geographical diversity of disadvantaged populations was explored in order to investigate whether interventions were more equitably distributed in those towns with more clustered population groups. Within each town, the geographical distribution of each population of need was mapped, and a Global Moran’s I statistic was computed. This produced an index value on a scale of −1 to +1, where +1 indicates clustering of population groups and −1 indicates dispersion of population groups (0 indicates random distribution). Index scores were divided into tertiles and the mean targeting ratios were compared across tertiles by computing Kruskal-Wallis H statistics. The Kruskal-Wallis tests were chosen because the distribution of targeting ratios was positively skewed. In order to test for the potential presence of trade-offs in cases where the interventions were specifically targeted at more than one group, average targeting ratios were examined according to the number of population groups the intervention targeted, with the association again being tested using Kruskal-Wallis H statistics.

## Results

A total of 183 individual pieces of infrastructure that were either complete or in progress were identified across eight Healthy Towns. Of these, 80 (44%) were classified as ‘physical activity’ (e.g. green gyms and play areas), 59 (32%) as ‘active travel’ (e.g. walking maps and signed cycle routes), 39 (21%) as ‘food systems’ (e.g. community cafes and allotments) and 5 (3%) as ‘healthy lifestyle’ (e.g. advice centres and information trails). The most common types of intervention were outdoor play areas/green space (27%), walking/cycling mapping/signage (23%), food growing (18%) and walking/cycling routes (11%).

Table 3 shows the relationship between town size and per-capita funding according to the number of people in each town and those within a ten minute walk. Greater overall funding was associated with lower per-capita funding for the majority of population groups.

**Table 3:** Per capita funding from the healthy towns programme

**Funding by town size group**			
	**Small**	**Medium**	**Large**
Mean funding per town (£M)	1.05	3.07	4.68
Funding per capita (£)^a^			
All people	59	20	14
All people with good access	63	29	43
BME	1538	240	96
BME with good access	1590	288	406
Child	237	79	58
Child with good access	249	116	172
Retired	666	203	183
Retired with good access	693	299	504
Socio-economic disadvantage	272	95	68
Socio-economic disadvantage with good access	283	134	196

^a^Values are per capita except those for retired populations, which are per household. Funding per capita refers to the money spent divided by the number of people in each population group. Good access refers to the number of these people that live within a ten minute walk of new infrastructure. *BME* Black and minority ethnic. *£* pounds sterling. *£M* pounds sterling in millions.

**Figure 2:**
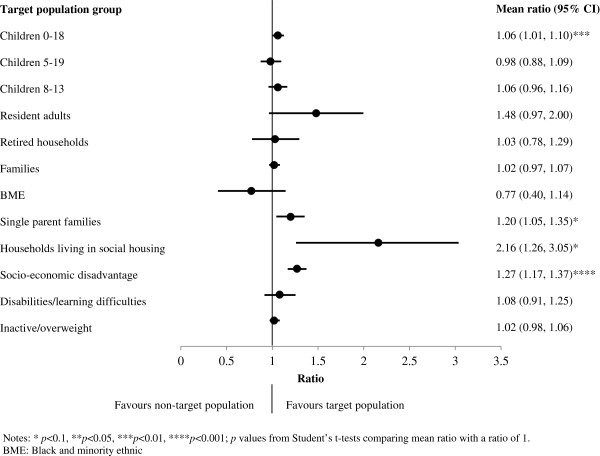
Mean targeting ratios by target population group.

**Figure 3:**
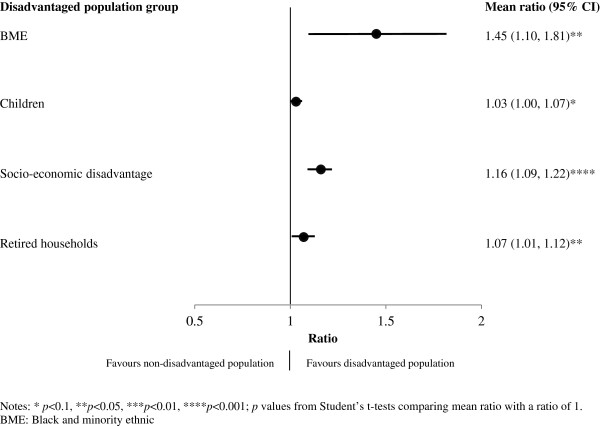
Mean targeting ratios by disadvantaged population group.

Figure 2 shows the target population group of each town along with the associated average targeting ratio. For all population groups except BMEs and children aged 5–19, ratios were above unity suggesting that infrastructure tended to be positioned in areas where the associated target population group lived. However, few of the estimated targeting ratios were statistically significant. While the targeting ratio for interventions targeted at socio-economically disadvantaged populations showed the highest statistical significance (targeting ratio 1.27, 95% CI 1.17 to 1.37, p<0.001), the largest targeting ratios were observed for social housing households (2.16, 95% CI 1.26 to 3.05) and resident adults (1.48, 95% CI 0.97 to 2.00). When targeting ratios were examined in relation to the four identified generally disadvantaged population groups (Figure 3), the ratios were all above unity, suggesting that the locations of infrastructure tended to favour these groups even if they were not necessarily the target population. Indeed, a comparison with Figure 2 shows that BMEs were more favoured overall (1.45, 95% CI 1.10 to 1.81) than for infrastructure specifically targeted at them (0.77, 95% CI 0.40 to 1.14).

Analysis of the mean targeting ratios according to intervention type and category showed that the majority of average ratios were above unity, suggesting that these populations were generally well-served by the items of infrastructure, although most did not reach statistical significance. For a full table of results, please see Additional files 1 and 2.

There was no evidence that overall population clustering was associated with targeting ratios (Table 4). However, when disaggregated by population group, there was evidence that targeting ratios were lowest in the most clustered populations for BME populations, whilst for socio-economically disadvantaged populations the highest ratio was observed in the most clustered tertile.

**Table 4:** Mean rank of targeting ratios for disadvantaged populations by tertiles of clustering

**Group**	**Tertile 1 (least clustered)**	**Tertile 2**	**Tertile 3 (most clustered)**	***p*value**^**a**^
All	359.9	367.5	371.5	0.83
BME	111.2	96.9	72.3	<0.001
Children	82.1	98.3	96.3	0.18
Socio-economic disadvantage	85.7	73.5	111	<0.001
Retired households	81.4	98.2	94.1	0.2

Notes: ^a^*p* values from Kruskal-Wallis test comparing mean rank with the median rank. *BME* Black and minority ethnic.

The average targeting ratio, according to mean rank, was significantly associated with the number of target groups for each intervention (p = 0.003), although the highest mean rank (139) was found for interventions targeted at the largest number of different groups (six), suggesting that multi-target population interventions tended to be better rather than more poorly targeted (full results not shown).

## Discussion

### Summary of main findings

Our findings suggest that infrastructure developed within each Healthy Town generally met our criterion (the targeting ratio) for spatial equity and this was independent of the amount of funding received. This was most statistically significant for socio-economically disadvantaged populations, which is in keeping with the evidence that some of the towns specifically identified generally disadvantaged areas to be targeted in their project initiation documents. We suggest that certain types of intervention may be less easy to locate with respect to populations who may particularly need them due to the nature of the resources they require. An example is food growing and food systems, reflecting limitations caused by the need for suitable land for these types of infrastructure programme. Spatial clustering of population groups was not associated with the success of spatial targeting in general, although areas with the high concentrations of socio-economically disadvantaged groups did experience highest targeting success. This illustrates how infrastructure providers can face particular challenges in areas where populations of need are not concentrated in particular places. We found no evidence that interventions targeted at more than one population group were less well located.

Examining where interventions were located in relation to who they were aimed at and the local context, as recommended by UK guidance [34,35], suggested that the Healthy Towns intervention was operating as it was initially intended in this respect. Thus, incorporating spatial equity analysis into the process evaluation of an environmental intervention allowed us to examine if the resources were directed to the most appropriate locations, a question which is appropriate to the current early stages of an intervention such as the Healthy Towns programme [31]. This is important in the context of evaluating environmental interventions, as health inequalities (and therefore population need) vary spatially [53] and therefore need careful spatial planning to ensure intervention success. The findings from this initial analysis may inform subsequent evaluation stages, providing explanation for outcomes, impacts and costs/benefits that may otherwise not be detected: our findings suggest that if the Healthy Towns programme is not successful and health inequalities are not reduced, it will be for reasons other than poor spatial planning. Indeed, qualitative evaluation of the implementation of the Healthy Towns programme has suggested that these reasons may include insufficient time, lack of evidence and poor alignment with national priorities [54]. We have generated new knowledge in the form of explicit, transparent and accurate information about the locating of infrastructure, thereby improving the evidence base for decision-making [30].

### Strengths and limitations

This study is novel in that it has demonstrated one approach to incorporating spatial equity analysis into the process evaluation of a complex environmental intervention. Identifying the spatial distribution of newly-built infrastructure allowed us to examine how access to new facilities was patterned according to population need. A particular strength was having access to a complete, up-to-date and detailed database of the location and progress of the interventions, constructed by consulting a wide range of sources and relevant programme staff. This was important as some of the pieces of infrastructure considered differed from those described in the original plans in the implementation documentation. All information that was not in a geographically referenced digital format was manually digitised. The availability of national, detailed geographic data (OS mapping, census statistics) combined with information obtained from the local areas allowed accurate referencing of information. All geographical data was subsequently checked via discussions with programme staff in each of the local areas.

Our work has some limitations. Defining the area of an intervention required a number of assumptions. The definition of a ten minute walking distance to approximate ‘good’ access was based on distances commonly used in the research literature, although some people will walk further to reach certain amenities [55], whilst others will be less mobile. In reality accessibility will vary by these individual characteristics. Because we did not have information on the locations of pedestrian only cut-throughs, common in urban areas, we used straight line distances rather than network distances when calculating accessibility. As just 183 new pieces of infrastructure were funded from the Healthy Towns programme, the sample size was limited for statistical analysis, particularly stratified analysis. In addition, we had no information regarding the quality of the interventions. Our population data were taken from the most recent (2001) Census of Population in England and Wales but this does mean that they reflect the local population almost ten years prior to the introduction of the Healthy Towns infrastructure. Finally, we did not attempt to evaluate the success of the interventions in terms of how and by whom they were used, or any resulting impact on behavioural or health outcomes.

## Conclusions

### Interpretation and contribution to existing knowledge

Building on existing research in the field of spatial equity analysis, we have presented the development and findings of a new method to help understand the implementation of interventions designed to change the built environment to promote healthier behaviours. We have contributed to the field of process evaluation, providing a robust means of initial evaluation of interventions compatible with UK [34,35] and US [36] recommendations, that considers the locations of populations of need [17]. We have established that infrastructure provision from the Healthy Towns programme in England was generally spatially equitable in that it was located in areas of highest population need, suggesting that in contrast to the literature on environmental disamenities [14,16,56], disadvantaged populations do not necessarily lose out when environmental modifications are made. With careful planning and implementation of interventions, therefore, interventions need not necessarily result in deprivation amplification [11].

### Implications for policy, practice and research

We propose that this form of spatial equity analysis should be incorporated as the first step in future process evaluations for spatially planned interventions in health, especially where it can answer questions at an early stage in the evaluation at which impact analysis may be premature. It may thus be a useful part of a formative evaluation. Nevertheless, the constantly changing policy landscape, coupled with often short-term fixed funding horizons for intervention delivery brings particular methodological and practical challenges, necessitating fast action to assemble required datasets whilst key personnel are still in post. Awareness of these challenges will be crucial to the success of similar analyses in future intervention studies.

## Competing interests

The authors declare they have no actual or potential competing interests, financial or otherwise.

## Authors’ contributions

AD acquired the data, carried out the analysis and drafted the manuscript. AJ made substantial contributions to conception and design of the analysis, the interpretation of data, and helped to draft the manuscript. DO, MP, MW and SC have provided advice regarding the design and analyses, and have been involved in revising the manuscript critically for important intellectual content. All authors read and approved the final manuscript.

## Supplementary Material

Additional file 1 Target populations and mean targeting ratio, by type and category of intervention. Click here for file

Additional file 2 Disadvantaged populations and mean targeting ratio, by type and category of intervention. Click here for file
