# Supplementary material for: Using spatial equity analysis in the process evaluation of environmental interventions to tackle obesity: the healthy towns programme in England
Source: Int J Equity Health. 2013 Jun 17;12:43. doi: 10.1186/1475-9276-12-43 (PMC3693867; doi:10.1186/1475-9276-12-43)
Supplement: Additional file 1 — Target populations and mean targeting ratio, by type and category of intervention. [file 1475-9276-12-43-S1.pdf]

Additional file 1

Target populations and mean targeting ratio, by type and category of intervention

	Average ratio	BME[a]	Children 0-18	Retired households	Socio-economic disadvantage	Families	Children 5-19
Type							
Advice/information	0.61** [7][b] (0.42, 0.80)[c]	N/A[d]	0.59* [2] (0.50, 0.69)	N/A	0.72 [1]	0.49** [2] (0.47, 0.50)	N/A
Cafe/food co-ops	1.41 [7] (0.94, 1.88)	N/A	1.05 [1]	N/A	1.53 [1]	0.93 [1]	N/A
Facilities for cycling/walking	1.01 [20] (0.89, 1.14)	N/A	1.00 [5] (0.84, 1.17)	N/A	1.09 [5] (0.53, 1.65)	0.96 [5] (0.76, 1.15)	N/A
Food growing	1.21** [70] (1.07, 1.35)	0.77 [6] (0.32, 1.22)	1.08 [17] (0.98, 1.17)	1.05 [1]	1.42** [7] (1.20, 1.64)	1.06 [15] (0.91, 1.20)	1.11 [2] (0.89, 1.33)
Green gym/dance studio	1.04 [35] (0.96, 1.11)	N/A	0.99 [8] (0.88, 1.09)	N/A	1.12 [7] (0.76, 1.48)	0.97 [7] (0.84, 1.10)	0.82 [1]
Outdoor play area/green space	1.15** [155] (1.08, 1.21)	0.79 [1]	1.06 [48] (0.99, 1.14)	1.43 [1]	1.27** [35] (1.12, 1.43)	1.01 [25] (0.91, 1.12)	N/A
Walking/cycling routes	1.02 [37] (0.95, 1.09)	N/A	0.97 [14] (0.90, 1.03)	1.43 [1]	1.69 [1]	0.96 [10] (0.81, 1.11)	0.96 [4] (0.85, 1.08)
Walking/cycling mapping/signage	1.10** [118] (1.04, 1.15)	N/A	1.12** [33] (1.04, 1.20)	0.92 [7] (0.59, 1.24)	1.33* [15] (1.09, 1.57)	1.08 [33] (0.99, 1.17)	N/A
Category							
Active travel	1.09** [102] (1.04, 1.15)	N/A	1.09* [33] (1.02, 1.16)	0.90 [5] (0.39, 1.40)	1.60** [6] (1.29, 1.92)	1.09 [29] (0.99, 1.19)	0.96 [4] (0.85, 1.08)
Food systems	1.23** [78] (1.10, 1.36)	0.77 [6] (0.32, 1.22)	1.07 [18] (0.98, 1.17)	1.05 [1]	1.44** [8] (1.25, 1.62)	1.05 [16] (0.91, 1.18)	1.11 [2] (0.89, 1.33)
Healthy lifestyle	0.75* [11] (0.57, 0.93)	0.79 [1]	0.76 [3] (0.21, 1.32)	N/A	0.72 [1]	0.72 [3] (-0.03, 1.48)	N/A
Physical activity	1.11** [268] (1.06, 1.15)	N/A	1.05 [74] (0.99, 1.10)	1.20 [4] (0.83, 1.57)	1.22** [57] (1.11, 1.34)	0.99 [50] (0.92, 1.05)	0.82 [1]

Continued over...

(Continued)

	Children 8-13	Resident adults	Over 50s	Disabilities and/or learning difficulties	Inactive/overweight	Single parent families	Households living in social housing
Type							
Advice/information	N/A	N/A	N/A	N/A	1.01 [1]	0.38 [1]	N/A
Cafe/food co-ops	N/A	1.58 [5] (0.96, 2.19)	N/A	N/A	N/A	N/A	N/A
Facilities cycling/walking	N/A	N/A	N/A	N/A	1.01 [5] (0.83, 1.19)	N/A	N/A
Food growing	N/A	1.01 [1]	N/A	1.10 [7] (0.92, 1.29)	1.25** [3] (1.17, 1.32)	1.59** [2] (1.56, 1.61)	1.88 [9] (0.92, 2.85)
Green gym/dance studio	N/A	N/A	1.24 [1]	N/A	1.02 [9] (0.92, 1.12)	1.28 [2] (1.08, 1.48)	N/A
Outdoor play area/green space	1.06 [23] (0.96, 1.15)	N/A	N/A	0.89 [1]	1.05 [12] (0.93, 1.16)	1.40 [7] (1.08, 1.71)	3.38 [2] (-0.16, 6.92)
Walking/cycling routes	N/A	N/A	N/A	N/A	1.03 [6] (0.89, 1.18)	1.50 [1]	N/A
Walking/cycling mapping/signage	N/A	N/A	N/A	N/A	0.95 [15] (0.87, 1.03)	1.08 [15] (0.88, 1.28)	N/A
Category							
Active travel	N/A	N/A	N/A	N/A	0.97 [11] (0.88, 1.06)	1.25 [6] (0.90, 1.60)	N/A
Food systems	N/A	1.48 [6] (0.95, 2.02)	N/A	1.10 [7] (0.92, 1.29)	1.25** [3] (1.17, 1.32)	1.59** [2] (1.56, 1.61)	1.88 [9] (0.92, 2.85)
Healthy lifestyle	N/A	N/A	N/A	0.89 [1]	1.01 [1]	0.38 [1]	N/A
Physical activity	1.06 [23] (0.96, 1.15)	N/A	1.24 [1]	N/A	1.02 [36] (0.96, 1.07)	1.18* [19] (1.00, 1.37)	3.38 [2] (-0.16, 6.92)

Notes: [a]BME: Black and minority ethnic; [b][n]; [c](CIs 95% upper, lower); *p <0.05, ** p<0.01 values from Student's t-tests comparing mean ratio with a ratio of 1; [d]N/A - no infrastructure in category
