# Supplementary material for: Using spatial equity analysis in the process evaluation of environmental interventions to tackle obesity: the healthy towns programme in England
Source: Int J Equity Health. 2013 Jun 17;12:43. doi: 10.1186/1475-9276-12-43 (PMC3693867; doi:10.1186/1475-9276-12-43)
Supplement: Additional file 2 — Disadvantaged populations and mean targeting ratio, by type and category of intervention. [file 1475-9276-12-43-S2.pdf]

Additional file 2

Disadvantaged populations and mean targeting ratio, by type and category of intervention

	Average	n	BME[a]	Children	Retired households	Socio-economic disadvantage
Type						
Advice/information	1.06 [12][b] (0.73, 1.38)[c]	3	1.20 (0.02, 2.37)	0.61** (0.54, 0.69)	1.65* (1.26, 2.03)	0.77 (0.57, 0.97)
Cafe/food co-ops	1.06 [24] (0.84, 1.27)	6	1.50 (0.69, 2.30)	0.77 (0.53, 1.01)	1.03 (0.80, 1.26)	0.93 (0.59, 1.27)
Facilities for cycling/walking	1.12* [80] (1.02, 1.22)	20	1.12 (0.86, 1.38)	1.01 (0.91, 1.11)	1.23 (0.96, 1.50)	1.13 (0.96, 1.29)
Food growing	0.97 [132] (0.91, 1.02)	33	0.64** (0.53, 0.75)	1.06 (1.00, 1.12)	1.00 (0.90, 1.10)	1.17** (1.06, 1.29)
Green gym/dance studio	1.02 [40] (0.90, 1.15)	10	0.78 (0.44, 1.12)	1.01 (0.89, 1.12)	1.18 (0.90, 1.45)	1.13 (0.86, 1.40)
Outdoor play area/green space	1.33* [196] (1.05, 1.60)	49	2.03 (0.93, 3.12)	1.05 (0.98, 1.13)	1.02 (0.92, 1.11)	1.21** (1.08, 1.34)
Walking/cycling routes	1.02 [80] (0.93, 1.10)	20	1.07 (0.79, 1.35)	0.98 (0.92, 1.04)	1.05 (0.93, 1.18)	0.98 (0.79, 1.16)
Walking/cycling mapping/ signage	1.33** [168] (1.12, 1.55)	42	1.94* (1.11, 2.78)	1.09* (1.02, 1.17)	1.05 (0.92, 1.19)	1.25** (1.09, 1.41)
Category						
Active travel	1.23** [236] (1.11, 1.34)	59	1.54* (1.11, 1.97)	1.06* (1.00, 1.12)	1.12 (1.00, 1.24)	1.19** (1.06, 1.31)
Food systems	0.98 [156] (0.92, 1.04)	39	0.77** (0.61, 0.94)	1.01 (0.95, 1.08)	1.00 (0.92, 1.09)	1.13* (1.03, 1.24)
Healthy lifestyle	1.03 [20] (0.82, 1.24)	5	1.08 (0.53, 1.64)	0.71* (0.45, 0.97)	1.49 (1.02, 1.97)	0.84 (0.60, 1.09)
Physical activity	1.25** [320] (1.06, 1.43)	80	1.74* (1.00, 2.49)	1.04 (0.99, 1.09)	1.04 (0.96, 1.11)	1.17** (1.07, 1.27)

Notes: [a]BME: Black and minority ethnic; [b][n]; [c](CIs 95% upper, lower); *p <0.05, ** p<0.01 values from Student's t-tests comparing mean ratio with a ratio of 1
